# Associations between different types of delivery, empathy, aggression, impulsivity and school bullying in children attending public and private schools in Pereira (Colombia)

**DOI:** 10.1016/j.heliyon.2025.e42387

**Published:** 2025-01-30

**Authors:** Julio C. Sánchez, William Martínez, Andrés M. García, Andrés F. Ramírez, Heidy Y. Mesa, Alejandra Kafruni, Paula M. Herrera

**Affiliations:** Faculty of Health Sciences, Universidad Tecnológica de Pereira, Pereira, 660003, Colombia

**Keywords:** Oxytocin, Delivery, Social behaviour

## Abstract

This study aimed to correlate exposure to oxytocin during childbirth with behavioral determinants in teenage students. The Barratt Questionnaire (BQ), the Buss and Perry Aggression Questionnaire (BPAQ) and the Bryant Empathy Index (BEI), respectively measured impulsivity, aggression and empathy; the results were correlated with the roles of school bullying through the Velásquez and Pineda scale. Mothers were asked about birth circumstances.

A total of 401 students were included (mean age 12 ± 1 years, 53,9 % were male, 53,3 % were attending a public school). 41,9 % of students had exogenous oxytocin exposure, 40,1 % had physiological oxytocin exposure, and 18 % had no oxytocin exposure. Regarding bullying, 75,1 % of students were classified as observers, 14,2 % were classified as victims, 6 % were classified as intimidators and 4,7 % exhibited an indifferent role. The mean value of the BPAQ was 78 ± 19, for the BEI was 78 ± 10 and for the BQ was 60 ± 10; all values were considered high. There were no significant differences among the type of delivery, sex and bullying roles or the type of delivery, aggressiveness and impulsivity according to sex; however, males had significantly lower empathy scores. There was no significant association between the type of delivery and the risk of assuming a bullying role. A regression model showed a significant association between attending a private school and a lower risk of developing a victim or intimidator role. This study could contribute to a better understanding of the processes involved in behavioral and emotional outcomes after birth, which can help to design prevention strategies to address increasing mental health problems in youth. Furthermore, this study could help emphasize the importance of promoting physiological delivery and find evidence that helps the scientific community design new work to deepen the relationship between oxytocin and behavior.

## Highlights

• All children included in this study exhibited high levels of impulsivity, aggressiveness and empathy.
• Male children showed a lower level of empathy than female children.
• Children who attended private schools had a lower risk of developing victim or intimidator roles.
• There were no significant differences between the type of delivery and bullying roles.
• There were no significant differences between the type of delivery and levels of empathy, aggression or impulsivity.

## 1 Introduction

Labor and birth are some of the most critical events in life and involve neurological and endocrine mechanisms, mainly conditioned by oxytocin (OXT), which may determine social behavior [1]. OXT is a nonapeptide synthesized primarily in the hypothalamus, at the parvocellular paraventricular and supraoptic nuclei, and, in more limited amounts, in the bed nucleus of the stria terminals [2,3]. These neurons are connected to the neurohypophysis, where OXT is released into the bloodstream in response to several stimuli, including breastfeeding, delivery and stress. However, OXT may also be synthesized in peripheral tissues such as sexual organs, the placenta, the corpus luteum and the heart [4].

Conventionally, this hormone is known for influencing female reproductive functions during labor, uterine contractions, breastfeeding, and maternal care. In obstetric practice, synthetic OXT is administered as part of labor induction protocols to enhance uterine smooth muscle contractions [5,6]. However, importantly, some studies have found oxytocin in the hypothalamus and plasma in males, suggesting some other functions unrelated to labor or breastfeeding that have not yet been unveiled [3,7,8].

OXT has a dual role as a hormone and a neurotransmitter [9]. Oxytocinergic axonal projections from the hypothalamus reach the prefrontal cortex, amygdala, and nucleus accumbens, allegedly modulating social behavior [2,7]. The role of OXT in sexual behavior, feeding, social interaction, affiliative behavior, motor activity, fear, stress, and yawning has also been described [10].

Furthermore, OXT is one of the most critical peptides related to particular social relation regulations, such as trust, emotional attachment, aggression, and empathy [11]. Some studies have explored its role in controlling aggressive behavior [12], and it has been associated with improving social attitudes [13]. Additionally, OXT has been tested as a therapeutic option to treat some psychiatric disorders such as autism and to improve social behavior [14,15].

The OXT receptor (OXTR) is a 389 amino acid polypeptide that belongs to class I G–protein-coupled receptors [4]. The OXTR has been associated with various functions in the central nervous system [16]; the anxiolytic effects of OXT have been demonstrated in mice through OXTR expressed in serotonergic neurons [17].

OXT plays a role in mating, empathy, and social cohesion in prairie voles [18]. This rodent species shares the tendency to develop tight bonds with selective conspecifics with humans, expressed as the preference to spend more time with a close individual and not with a strange individual. In these animals, OXT increased OXTR methylation in the fetal brain when administered to mothers during labor, and oxytocin-exposed voles developed better social abilities [19].

On the other hand, OXT has also been associated with aggressive behaviors. Aggression is a universal behavior in humans and animals that appears in the race for resources, territory, mates, and, in other cases, dominance [20]. It is considered a multifactorial state defined as an activity through which a subject seeks to cause damage or pain to another individual willing to avoid it [21,22]. In humans, aggression has been associated with poor language skills to express inner states, so it is more easily observed in children and teenagers, individuals with some mental disorders and individuals with hostile social relations leading to self-damage and even suicide [23]. For these reasons, aggression is considered a risk factor for inducing delinquent and antisocial behaviors, thus becoming a social and public health problem [24,25]. OXT also more often directly affects and modulates aggressive reactions by decreasing aggression in men than in women [26].

Empathy is a cognitive and affective process that allows the emotional states of others to be understood. Its core components (motor, emotional, cognitive) are expressed in different developmental stages, from the initial emotional bond with parental authorities to the integration of social rules, and are associated with the socialization process, emotional intelligence, self-esteem, and lower antisocial and delinquency rates [27,28]. OXT has been related to the development of empathy, although the neurobiological mechanism of this relationship is not yet known [29].

Impulsivity has been defined as an action without forethought, low consciousness, and behavior in individuals with lower judgment and reflexivity than most individuals with equal thought [30]. The intranasal administration of OXT reduces impulsivity in subjects with specific OTXR genotypes [31].

Violent behavior is related directly to empathy, aggression, and impulsivity; their balance shapes social interactions. Bullying is defined as frequent, unwanted, and intentional aggressive behavior among individuals within a perceived or observed power imbalance [14]. Additionally, bullying could be defined as a set of hostile actions against an individual over some time, consisting of different situations of intimidation, harassment, abuse, and victimization among school children [25], which may have severe consequences for child health [32].

The concept of bullying is built into three dimensions: intention of harm, repetitiveness and power imbalance [33]. The effect of bullying is victimization (the repeated occurrence of harm performed by peers) with adverse outcomes in social development and academic performance [34]. Some challenges of the long-term effects of victimization are the disruption of peer networks, low self-esteem and internalizing behaviors that perdure in young adult cycle with more pervasive victimization interactions and relationships [35].

The understanding of bullying dynamics is a challenge to design policies and strategies with the objective of preventing its effects on infancy and adolescence. One of the approaches of bullying involves the definition of three-factors (victimization, symptomatology and intimidation) [36]. The bullying prevalence among female students is approximately 30 % and it could increase to 35 % in male students [37]. The forms of bullying between male students can be physical, verbal or symbolic violence, in the contrary, female students tend to act more indirectly in relational harassment expression such as spreading rumors or social exclusion between peers [38].

Centering all these concepts around the fact that the frequency of elective cesarean delivery, in which there is no fetal brain exposure to physiological OXT levels, and the systematic use of exogenous OXT to induce labor, in which the fetal brain is exposed to higher nonphysiological OXT levels, have substantially increased [39,40], this study aimed to determine to what extent OXT exposure during labor plays a role in the expression of aggression, empathy and impulsivity in school children and teenagers.

## 2 Methods

This study was conducted in public and private educational institutions in Pereira and Dosquebradas, two cities in Colombia. The study included 401 students from the sixth and seventh grades; the median age was 12 years, 54 % of the students were male, and 53 % were attending public schools.

This study was cross-sectional research with a convenience non-probabilistic sampling due to the barriers to accessing schools. The eligibility criteria were that the children and parents or proxies consented to participate and that parents could give information about the delivery conditions. The children verbally informed consent, and the parents or guardians provided written consent. The research team applied the tests in daytime sessions within the academic agenda of the participating schools in the presence of the lead teacher of each group. This study work has been carried out in accordance with The Code of Ethics of the World Medical Association (Declaration of Helsinki). Specific questionnaires were applied with previous approval by the institution's authorities. Parents or guardians signed informed consent forms, and students signed informed assent forms. The Bioethical Committee of the Universidad Tecnológica de Pereira approved this study (Code: CBE-SYR-162016).

### 2.1 Impulsivity

Impulsivity was measured with the Barratt Questionnaire. This questionnaire has been validated in multiple languages, including Spanish. This instrument consists of 29 questions and contains 3 subdimensions of impulsive behavior: attention (the capacity to be focused or vigilant, 8 questions), nonplanning (to act by living in the moment, 11 questions) and motor (acting following an impulse, without thinking, 10 questions) subdimensions [41]. The external validity assessment of the questionnaire through the confirmatory factor analysis is 0,95 with an appropriate result in the goodness-of-fit tests. The internal consistency reliability through Cronbach's alpha and omega of the questionnaire was 0,81 [42].

### 2.2 Aggressivity

Aggressivity was measured with the Buss and Perry Questionnaire, an instrument that has been validated in Spanish [43,44]. The instrument comprises four dimensions and 29 items: 11 items for physical aggression, 9 items for verbal aggression, 6 items for rage, and 3 items for hostility. The external validity assessment of the questionnaire through the confirmatory factor analysis is 0,99 with an appropriate result in the goodness-of-fit tests. The internal consistency reliability through Cronbach's alpha and omega of the questionnaire was 0,88 [45].

### 2.3 Empathy

For assessing empathy, the Bryant index [46], composed of 22 items, was used [47]. This instrument is divided into 13 empathic questions and 9 apathetic questions. The external validity assessment of the questionnaire through the confirmatory factor analysis is 0,98 with an appropriate result in the goodness-of-fit tests. The internal consistency reliability through Cronbach's alpha of the questionnaire was 0,872 [48].

### 2.4 Bullying

Bullying behavior was assessed with the Velasquez and Pineda scale, designed and validated in Spanish [49,50]. The scale consists of 35 items divided into three dimensions of aggression: physical, verbal, and social [49]. Its goal is to identify the predominant role of each individual, which can be indifferent, intimidator (9 questions), victim (12 questions) or observer (14 questions). The external validity assessment of the questionnaire through the confirmatory factor analysis is 0,96 with an appropriate result in the goodness-of-fit tests. The internal consistency reliability through Cronbach's alpha and omega of the questionnaire was 0,85 [51].

### 2.5 Birth delivery history

The participants’ parents were questioned about the clinical birth and pregnancy history, whether a cesarean section was performed, and whether exogenous oxytocin was needed to induce labor; the students were grouped according to these medical records, and a digital database was constructed.

### 2.6 Statistical analysis

Statistical analyses were performed using IBM software SPSS Statistics version 28.0 (SPPS Science, Chicago, IL, USA) and R 4.2.1. Data were assessed for normality through a Shapiro‒Wilk test. Then, unpaired t tests were used to evaluate the differences between study groups, and p < 0.05 was considered significant. Odds ratios (ORs) were estimated to assess the association between types of delivery and certain types of bullying roles. The estimates were significant if the confidence intervals (CIs) of the ORs did not include the null value. Logistic regression was performed to determine the interaction between the type of delivery and confounders and a specific type of bullying role.

## 3 Results

A total of 401 junior high school students were included in the study; the mean ± SD (standard deviation) age was 12 ± 1 years, and 53,9 % of the students were male. In Table 1, the data are presented by type of school. The pregnancy delivery routes were as follows: 168 (41,9 %) involved oxytocin administration, 161 (40,1 %) involved physiological oxytocin exposure and 72 (18 %) involved elective cesarean delivery (ECD) without oxytocin exposure. A total of 214 (53,3 %) students were attending a public school, and 187 were attending a private school. For the bullying role, 75,1 % of the students were classified as observers of bullying, 14,2 % were classified as victims, 6 % were classified as intimidators and 4,7 % exhibited an indifferent role. The mean ± SD value of the Buss and Perry Questionnaire for aggressive behavior was 78 ± 19, that for the Bryant Empathy Index was 78 ± 10 and that for the Barratt Impulsiveness Scale was 60 ± 10.

**Table 1:** Bullying role and behavior patterns by type of school.

Variables		Private	Public
Bullying role	Observer	136 (72,7 %)	165 (77,1 %)
	Victim	26 (13,9 %)	31 (14,5 %)
	Intimidator	9 (4,8 %)	15 (7 %)
	Indifferent	16 (8,6 %)	3 (1,4 %)
Impulsive behavior^a^		59 ± 10	60 ± 10
Aggressive behavior^a^		77 ± 19	79 ± 19
Empathy^a^		76 ± 10	79 ± 10

a The data is presented in mean ± SD SD: Standard Deviation.

There was no significant difference between the type of delivery and the scores obtained on the Buss and Perry Questionnaire, Bryant Empathy Index, and Barratt Impulsiveness Scale determined through an unpaired *t*-test. In Table 2, the data are presented by type of delivery.

**Table 2:** Bullying roles and behavior patterns by type of delivery.

Variables		Physiological	ECD	Oxytocin administration
Bullying role	Observer	126 (78,3 %)	49 (68,1 %)	126 (75 %)
	Victim	20 (12,4 %)	12(16,7 %)	25 (14,9 %)
	Intimidator	7 (4,3 %)	5 (6,9 %)	12 (7,1 %)
	Indifferent	8 (5 %)	6 (8,3 %)	5 (3 %)
Impulsive behavior^a^		59 ± 9	60 ± 8	61 ± 10
Aggressive behavior^a^		77 ± 20	76 ± 19	79 ± 19
Empathy^a^		78 ± 10	78 ± 10	77 ± 10

a The data is presented in mean ± SD SD: Standard Deviation ECD: Elective Cesarean Delivery.

There was a significant difference between Bryant Empathy Index scores obtained by sex, with male students having lower scores (males, 74 ± 10 vs. females, 81 ± 9; p < 0.0001, obtained by an unpaired t-student test). There were no differences regarding aggressivity and impulsivity by sex. In Table 3, the data are presented by type of delivery.

**Table 3:** Bullying roles and behavior patterns by sex.

Variables		Male	Female
Bullying role	Observer	163 (75,5 %)	138 74,6 %)
	Victim	27 (12,5 %)	30 (16,2 %)
	Intimidator	15 (6,9 %)	9 (4,9 %)
	Indifferent	11 (5,1 %)	8 (4,3 %)
Impulsive behavior^a^		59 ± 9	60 ± 10
Aggressive behavior^a^		77 ± 19	79 ± 19
Empathy^a^		74 ± 10	81 ± 9

a The data is presented in mean ± SD SD: Standard Deviation.

A risk analysis was performed to assess the relationship between the type of delivery and specific bullying roles. For the intimidator role and the victim role, a positive outcome was defined as being classified as an intimidator or a victim, and a negative outcome was defined as being classified as having any other role. The exposure was defined as positive if the delivery was an ECD or involved oxytocin administration and as negative if the delivery involved physiological exposure. In Table 4, the odds ratios are presented, but there was no significant association between the type of delivery and the risk of developing a specific type of bullying role.

**Table 4:** Estimated odds ratio for bullying role by type of delivery.

Bullying role	Analysis group	OR	CI 95 % Limits
Intimidator	(ECD vs Physiological)	1,64	(0,50–5,35)
	(Oxytocin administration vs Physiological)	1,66	(0,63–4,32)
Victim	(ECD vs Physiological)	0,56	(0,25–1,24)
	(Oxytocin administration vs Physiological)	0,81	(0,43–1,52)

OR: Odds Ratio CI: Confidence Interval ECD: Elective Cesarean Delivery.

A polynomial logistic regression model was performed to further assess the interaction between the covariates and the probability of developing a specific type of bullying role. The regression showed a significant association between attending a private school and a lower risk of developing a victim (β = −1.80 (0.69), p = 0.009, OR 0.16 (0.04–0.63) or an intimidator role (β = −2.20 (0.76), p = 0.004, OR 0.11 (0.02–0.49), and there was no significant association of the type of delivery and sex with the bullying role. In Table 5, the regression model is presented.

**Table 5:** Polynomial logistic regression model for bullying role by type of school, type of delivery and sex∗.

Bullying role		B	SE	Wald	Sig.	Exp(B)	CI 95 %	
							Lower Limit	Upper Limit
Observer	Intercept	4449	0,747	35,51	0,000			
	Private school	−1825	0,643	8,04	**0,005∗**	0,161	0,046	0,569
	PD	−0,505	0,591	0,731	0,392	0,603	0,190	1920
	ECD	−1002	0,637	2,47	0,116	0,367	0,105	1281
	Male	-,036	0,489	0,005	0,941	0,964	0,370	2515
Victim	Intercept	2960	0,782	14,32	0,000			
	Private school	−1807	0,687	6918	**0,009∗**	0,164	0,043	0,631
	PD	−0,725	0,651	1241	0,265	0,484	0,135	1734
	ECD	−0,795	0,709	1258	0,262	0,452	0,113	1812
	Male	−0,305	0,544	0,315	0,575	0,737	0,254	2140
Intimidator	Intercept	2030	0,846	5753	0,016			
	Private school	−2203	0,763	8347	**0,004∗**	0,110	0,025	0,492
	PD	−1057	0,751	1983	0,159	0,347	0,080	1513
	ECD	−0,900	0,818	1210	0,271	0,407	0,082	2020
	Male	0,347	0,639	0,294	0,587	1415	0,404	4952

CI: Confidence Interval PD: Physiological Delivery ECD: Elective Cesarean Delivery ∗ *p* < 0.05.

## 4 Discussion

This study assessed the relationship between the type of delivery related to oxytocin exposure and aggressive, impulsive, and empathic behavior patterns in junior high school students in Colombia. An exploration of the risk for developing a specific type of bullying role and differences in behavior by sex and type of institution were also assessed.

Intrapartum synthetic oxytocin has been widely used to prevent postpartum hemorrhage and induce labor [52]. Several studies have assessed the risk of this exposure on mental health [53,54]. In animal models, intranasal administration of oxytocin is associated with disruption of the hypothalamic-pituitary-adrenal (HPA) axis and social behavior dysfunction associated with isolation patterns [55]. Additionally, intrapartum oxytocin exposure (IOS) has been correlated with lower APGAR scores and global motor development [56]. There are reports of an association between higher IOS and cognitive impairment in children [57]. An analysis of postpartum outcomes of women exposed to IOS showed an association with postnatal depressive symptoms, later associated with suboptimal mother-to-infant bonding and, therefore, with poorer emotional and behavioral development in children [58].

Nevertheless, other studies have not found any association between labor induction and behavioral or emotional development in children.

Conversely, ECD increases the odds of having a psychological and behavioral impairment in early childhood and a greater risk of developing hematological malignancies and respiratory problems [59]. In Brazil, there was a higher prevalence of emotional/behavior problems in preschoolers with poor peer interactions and an increased risk for attention-deficit/hyperactivity disorder (ADHD) and autism spectrum disorders (ASDs) in children born by ECD [60].

In the present study, the data showed no association between the different types of delivery and students’ empathy, impulsiveness or aggressive behavior scores. The estimated ORs showed no increased risk for victim or intimidator roles for nonphysiological deliveries. However, the regression model showed a protective association between enrollment in a private school and the development of an intimidator or victim role.

In the assessment by sex, males exhibited lower scores on the Bryant Empathy Index; all the other scores showed no differences regarding sex. Testosterone has been traditionally associated with fewer empathic interactions obtained from the observations of children with ASD, and antenatal exposure to androgens has been linked to the suppression of social cognition [61]. However, recent studies have shown that this link does not have sufficient evidence [62]. Therefore, the lower scores obtained in the empathy index in this study are probably related to additional social, familial and individual factors that were not assessed and cannot support an androgen-related empathy suppression hypothesis [63].

The predictors for intimidator or victim bullying roles in children are parental punitive and persuasive behaviors, education level, and alcohol abuse, which are outcomes of ACEs and poor socioeconomic conditions [64,65]. In this context, attending a private school could be analyzed as a protective factor because it is associated with better economic, social and educational conditions for parents to enroll their child in this type of institution [64]. The difference between private and public school environments could also be a factor in explaining this finding since, at least in Colombian schools, there are significant differences in location, facility quality, the availability of educational resources, teacher qualifications, orderliness and administrative efficiency and effectiveness [66]. These circumstances mean that the results presented here do not apply to all school children populations. However, they can help understand the relationships between biology and social behavior.All these interactions contribute to a significant risk for behavioral problems with a biological predisposition due to altered intrapartum oxytocin exposure [57]. This study did not obtain information about oxytocin doses or administration times because we had no access to clinical records, which is the main limitation of the results presented here. Another important limitation is that this study obtained the clinical information from the mother's self-report, which can constitute a bias because it trusts in the recalls of the delivery circumstances, which can be variable since delivery could transit from oxytocin-induced labor to cesarean delivery, which makes challenging this assessment.

The link between a mother's ACE-exposed oxytocin-modified response and the intrapartum physiological effects on her child is unknown. However, a better understanding of the biological processes involved in children's behavioral and emotional outcomes could help design prevention strategies and identify therapeutic targets to address the increasing mental health problems and deteriorating mental health in youth.

In conclusion, all children in this study exhibited high levels of impulsivity, aggressiveness, and empathy. Additionally, male children showed a lower level of empathy than female children. Furthermore, private school children had a lower risk of developing victim or intimidator roles. This study could not find significant differences between the type of delivery and bullying roles or between the type of delivery and levels of empathy, aggression, or impulsivity. This study could contribute to a better understanding of the processes involved in behavioral and emotional outcomes after birth, which can help to design prevention strategies to address increasing mental health problems in youth. Furthermore, this study emphasizes the importance of promoting physiological delivery and finding evidence that helps the scientific community design new work to deepen the relationship between oxytocin and behavior.

## CRediT authorship contribution statement

**Julio C. Sánchez:** Writing – review & editing, Writing – original draft, Validation, Supervision, Project administration, Methodology, Investigation, Funding acquisition, Formal analysis, Data curation, Conceptualization. **William Martínez:** Writing – review & editing, Methodology, Investigation, Formal analysis. **Andrés M. García:** Writing – review & editing, Methodology, Investigation, Formal analysis, Data curation. **Andrés F. Ramírez:** Writing – review & editing, Methodology, Investigation, Formal analysis. **Heidy Y. Mesa:** Writing – review & editing, Methodology, Investigation, Formal analysis. **Alejandra Kafruni:** Writing – review & editing, Methodology, Investigation, Formal analysis. **Paula M. Herrera:** Writing – review & editing, Validation, Supervision, Investigation, Conceptualization.

## 5 Ethics statement

The Bioethical Committee of the Universidad Tecnológica de Pereira approved this study (Approval number: CBE-SYR-162016). Informed consent was obtained from the parents or guardians for the participation and publication of any data included in the manuscript. Students signed informed assent forms. This study complies with all ethical regulations stablished in the Code of Ethics of the World Medical Association (Declaration of Helsinki).

## Data and Code availability statement

Data will be made available on request. For requesting data, please write to the corresponding author.

## Funding

The 10.13039/501100013367Technological University of Pereira supported this research financially. Grant number: 5-18-14.

## Declaration of competing interest

The authors declare that they have no known competing financial interests or personal relationships that could have appeared to influence the work reported in this paper.
